# Extensive Destruction of the Soft Parts near Angle of the Mouth from Sloughing after Fever

**Published:** 1875-02

**Authors:** Robert T. Godfrey

**Affiliations:** Professor of Surgery, University of Bishop's College, Lennoxville, Attending Surgeon, Montreal General Hospital.


### ARTICLE V.

### *Extensive Destruction of the Soft Parts near the Angle of the Mouth, from Sloughing after Fever. Plastic Operation; Recovery.*

BY ROBERT T. GODFREY, M. D.,

Professor of Surgery, University of Bishop's College, Lennoxville, Attending Surgeon, Montreal General Hospital.

The following case was admitted into the Montreal General Hospital on the 13th July, 1874, and as it illustrates the beneficial results of plastic surgery, I deem it of sufficient interest to lay before your readers. The history as given by the patient himself is as follows:

William Bouchet, æt. 19, a French Canadian: a strong, well proportioned young man,—some two years ago suffered from an attack of "Typhoid Fever." He was ill for several weeks. In the course of his illness, and towards the close of the fever, his face became inflamed, he suffered much pain of a burning character, soon an ulcerated spot occurred, and the whole of the cheek separated and fell out; subsequently his teeth loosened, both in the upper and lower jaw, and were taken away by his doctor, together with several pieces of bone; he made a very slow recovery. Upon examination I ascertained that nearly the whole cheek on the left side had sloughed. The buccal cavity was completely absent. The molar teeth in the upper and lower jaw were gone, as well as a large portion of the alveolar processes. The integument was adherent to the bones of the face, and there existed a large opening the size of a crown piece, through which could be seen the tongue and the soft palate. The motion of the jaw was limited, and although he was quite able to pass into his mouth a good sized ball of food he could not masticate properly. He experienced great difficulty in guiding his food, when masticated, into the pharynx, as it would pass out through the opening unless prevented by his hand. This rendered him very miserable, as anything like liquid food would run down his jaw and the side of his neck, and occasionally produce excoriations. The lips were entire; the angle of the left upper lip was curved upwards and inwards and was attached to the superior maxilla, while the lower lip curved downwards and was attached to the inferior maxilla. At the upper and outer part of the gap there exsted an opening into the antrum, which was quite visible. Looking at him from the injured side his appearance was very revolting. The poor fellow had to keep the part covered with a pad of linen to prevent the saliva from flowing down the side of his face and neck.

On the 21st of July I performed the following operation. The patient was placed under the influence of chloroform, and with the able assistance of my colleague, Dr. McCallum, I commenced by paring very freely the edges of the aperture. The integuments were then separated from their attachments to the superior and inferior jaw bones. The angle of the upper lip was freed, and the lip itself separated from its attachment to the incisive fossa; this gave ample room and the lip came down to its natural position without any dragging or strain on the nose. A large flap, semilunar in shape, was then taken from the integument situated over the body of the inferior maxilla and submaxillary space; this was perfectly freed and came up and fitted the aperture. The angle of the flap, somewhat V shaped, filled a similar shaped space which was left after turning the upper lid downwards. The angle of the mouth and the angle of the flap were fixed in their places by harelip needles with the figure of eight suture. The edges of the rest of the wound were retained in situ by wire sutures. The flaps were so freely separated from their busy attachments that there was no straining of the parts, which greatly conduced to the success of the operation. On the fourth day the needles were removed, the sutures were, however, allowed to remain. He had on the following day a slight attack of erysipelas, which was relieved by the usual means. From this time all progressed favorably.

The sutures were not removed until firm union had taken place. The patient left the hospital on the 8th of August, eighteen days after the operation. The accompanying engravings are from photographs by Notman, and give a most faithful representation of the appearance of the patient both before and after the operation.

---

### EDITORIAL, ETC.

